# Deep Insight into the Phosphatomes of Parasitic Protozoa and a Web Resource ProtozPhosDB

**DOI:** 10.1371/journal.pone.0167594

**Published:** 2016-12-08

**Authors:** Tamanna Anwar, Samudrala Gourinath

**Affiliations:** School of Life Sciences, Jawaharlal Nehru University, New Delhi, India; Centro de Investigacion y de Estudios Avanzados del Instituto Politecnico Nacional, MEXICO

## Abstract

Phosphorylation dynamically regulates the function of proteins by maintaining a balance between protein kinase and phosphatase activity. A comprehensive understanding of the role phosphatases in cellular signaling is lacking in case of protozoans of medical and veterinary importance worldwide. The drugs used to treat protozoal diseases have many undesired effects and the development of resistance, highlights the need for new effective and safer antiprotozoal agents. In the present study we have analyzed phosphatomes of 15 protozoans of medical significance. We identified ~2000 phosphatases, out of which 21% are uncharacterized proteins. A significant positive correlation between phosphatome and proteome size was observed except for *E*. *histolytica*, having highest density of phosphatases irrespective of its proteome size. A difference in the number of phosphatases among different genera shows the variation in the signaling pathways they are involved in. The phosphatome of parasites is dominated by ser/thr phosphatases contrary to the vertebrate host dominated by tyrosine phosphatases. Phosphatases were widely distributed throughout the cell suggesting physiological adaptation of the parasite to regulate its host. 20% to 45% phosphatome of different protozoa consists of ectophosphatases, i.e. crucial for the survival of parasites. A database and a webserver “ProtozPhosDB” can be used to explore the phosphatomes of protozoans of medical significance.

## Introduction

Protozoan infection is a serious health problem in poor and developing countries with poor sanitary conditions. Protozoa are single-celled, microscopic organisms that can live freely or parasitic in nature. Parasitic protozoa are capable of multiplying in mammals, which contributes to their survival and also causes serious infections to develop from just a single organism. Protein phosphorylation and dephosphorylation are integral mechanism implicated in the regulation of cellular activities in any organism. Enzymes belonging to the class kinases and phosphatases that act antagonistically control the reversible phosphorylation. Protozoan parasites are capable of sustaining in diverse environmental conditions by exploiting the host’s surface proteins through phosphorylation/dephosphorylation of ser/thr and tyr residues, that helps in cell growth inhibition and parasite-host interaction [1]. Kinases have been the focus of research forefront since long, but the emerging evidence suggests that phosphatases also play critical role in signaling network and cell-fate decisions. The versatility of phosphatase action emphasizes the need for understanding the network complexity of the signaling pathway in protozoan parasites as phosphatases are becoming extremely important both at research forefront and as targets for drug development. In this study, we have analyzed the phosphatomes of protozoan parasites belonging to the phyla Apicomplexa, Kinetoplastida and *Entamoeba histolytica*, *Giardia lamblia*, *Trichomonas vaginalis* and a microsporidium *Encephalitozoon cuniculi*, these are responsible for important diseases like Chagas' disease, giardiasis, leishmaniasis, amoebiasis, trichomoniasis, and sleeping sickness. The drugs used to treat these diseases are turning useless due to the identification of new drug-resistant strains. Moreover, prolonged treatments pose sever side effects [2, 3]. To reduce the effect of protozoan infections and dysentery, new drugs that preserve therapeutic efficiency and are far from side effects, are desired. Therefore, it is critical to search for more effective drug targets to treat infectious diseases caused by protozoan parasites.

Phosphorylation is critical for the development of the parasites including protozoan parasites. A bulk of data is available for kinases, on contrary, less has been known for phosphatases in unicellular parasites. The availability of full genomic sequences of several protozoan parasites has led this study to provide the first account of our current understanding of phosphatases in parasitic protozoans.

## Materials and Methods

We performed preliminary analysis of the phosphatomes of several species of unicellular parasites that belong to Apicomplexa: *Plasmodium falciparum* (*Pf*); *Plasmodium vivax* (*Pv*); *Cryptosporidium pervum* (*Cp*); *Babesia bovis* (*Bb*), *Theileria parva* (*Tp*), *Toxoplasma gondii* (*Tg*), as well as Kinetoplastids: *Trypanosoma brucei* (*Tb*), *Trypanosoma cruzi* (*Tc*) and *Leishmania major* (*Lm*), *Entamoeba histolytica* (*Eh*), *Giardia lamblia* (*Gl*), *Trichomonas vaginalis* (*Tv*), a microsporidium *Encephalitozoon cuniculi* (*Ec*) and *Homo sapiens (Hs)*. The complete set of predicted protein sequences from the ORFs of the selected protozoa genome has been obtained from UniProt release 2014_01 [4]. Phosphatases are classified based on catalytic signature motifs and structural domains, a deeper classification of phosphatases is mentioned in our previous work on *E*. *histolytica* and others [5–7].

The program CD-HIT was used to remove redundant sequences [8]. A sensitive sequence analysis method was used for analyzing the genomes of the above mentioned protozoans for PPs. Standalone version of InterProScan5 was used to make domain assignments for PP catalytic domain containing gene products. Each of the phosphatase domain containing proteins in all the protozoans were queried against the 18347 protein families and 7512 domains available in the InterPro database (InterPro 50.0) [9]. InterPro is a powerful tool for classification and function prediction of protein sequence. Protein sequences with phosphatase domain having e-value score of 10^−4^ were selected for the analysis. CELLO v.2.5 [10] was used for subcellular localization prediction. Structural domain analysis was carried out using Phyre2 (Protein Homology/AnalogY Recognition Engine) that is a web-based service for protein structure prediction [11]. To identify the molecular function, biological process and pathway of each protein, the PANTHER Classification System was used [12]. All the data obtained in the analysis of 15 parasitic protozoans was integrated into the database ProtozPhosDB. Venn diagram was made using InteractiVenn [13].

ProtozPhosDB is developed by using MySQL database software application along with PHP. For creating web interphase, Bootstrap template was used, AJAX and JQuery were deployed to make the database application more interactive. The website framework is quite portable and it can be easily transformed with any other list of proteins.

## Results and Discussion

### 1.1 ProtozPhosDB

ProtozPhosDB is a public repository dedicated to the phosphatases encoded by completely sequenced parasitic protozoans. It is an on-line web portal developed to assist scientists in obtaining information about phosphatases in parasitic protozoans belonging to the phyla Apicomplexa, Kinetoplastida and few others with completed genome sequence. The web interphase of ProtozPhosDB can be dynamically searched for phospahatses belonging to different families and sub-families or by their localization. There are three different types of searching from database;

1. Simple search: It can be done to search for any type phosphatase by entering keywords. The user should enter keyword (serine, threonine, tyrosine, etc.) in the search textbox then select search anywhere and hit on Go. This will output all the phosphatases of the searched family in all the fifteen protozoans. The option “match exactly” can be used to search for phosphatases from particular family (e.g. protein serine/threonine phosphatase, protein tyrosine phosphatase, etc.).
2. Advance search: In advanced search the user can retrieve the phoaphatases in any of the fifteen parasitic protozoans by selecting the organism from the organism dropdown menu then from the family dropdown menu select the family of phosphatase and then sub-family from the sub-family dropdown menu and hit search button. The user can also search for all the phosphatases in a particular organism by selecting all from family and sub-family dropdown menu.
3. Search by localization: The database can also be searched according to localization of phosphatases. Here also the user has to select organism from the organism dropdown menu and then localization from the localization dropdown menu and hit search.

The results are displayed in the form of table indicating total number of entries. The results provide information regarding all the phosphatases of the selected family, the 3D structural domain, sub-cellular localization, molecular function/process and pathway annotation. The information regarding phosphatases in fifteen protozoans of medical importance is organized in single database to expedite retrieval of information regarding phosphatases.

### 1.2 Protozoan Parasite Proteome, Kinome and Phosphatome

**Fig 1:**
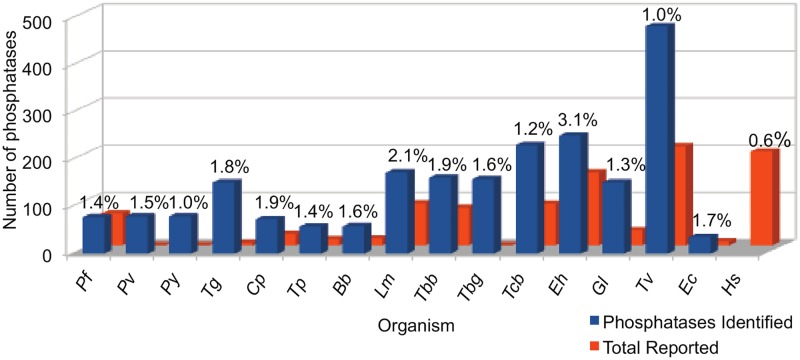
Bar diagram-representing comparison of phosphatases in the genomes of parasitic protozoa and human. Orange bars represents the number of phosphatases reported in previous studies. The percentage of phosphatase genes in the proteome complement is provided against every bar.

Kinomes and phosphatomes of several of these unicellular parasites have been analyzed in earlier studies [14–20]. Comparison of phosphatomes (phosphatases identified in the present study) and kinomes of the selected unicellular parasites (Table 1) shows that phosphatomes of Apicomplexa is a bit larger than their kinome sizes except for *Pf* where kinome is slightly larger. In case of *Eh*, *Gl*, *Tv and* Kinetoplastids number of kinases is much larger than phosphatases, except for *Tc* (Table 1). We have identified larger number of phosphatases in selected unicellular parasites than reported (Fig 1). Number of kinases and phosphatases reported in earlier studies in these organisms is given in Table 1. Study of phosphatome in of *Pv*, *Py* and *Tbg* is reported for the first time in the present analysis, while in case of *Tg* and *Ec* very few phosphatases have been reported earlier.

**Table 1:** Comparison of proteome, kinome and phosphatome of protozoan parasites.

Organism	Uniprot Id	Proteome Size	Phosphatome Size Present Study	Phosphatome Size Reported	Kinome Size Reported
*Apicomplexa*					
*Cp*	353152	3805	73	24 [14, 15]	61 [19]
*Tg*	5811	8404	151	5 [14, 15]	135 [19]
*Bb*	5865	3960	58	14 [14, 15]	35 [19]
*Tp*	5875	4071	57	13 [14, 15]	38 [19]
*Py*	73239	7757	79	-	63 [19]
*Pv*	126793	5389	79	-	68 [19]
*Pf*	36329	5353	77	67 [16]	89 [19]
*Kinetoplastida*					
*Lm*	5664	8038	172	89 [14, 15]	199 [20]
*Tbg*	679716	9668	158	-	-
*Tbb*	1185	8587	161	79 [14, 15]	176 [20]
*Tcb*	353153	19242	230	88 [14, 15]	190 [20]
*Eh*	5759	7959	250	144 [14, 15]	331 [19]
*Tv*	5722	50191	482	210 [14, 15]	931 [19]
*Gl*	5741	11207	151	32 [14, 15]	278 [17]
*Ec*	284813	2008	35	8 [14, 15]	32 [19]

### 1.3 Comparative Analysis of Protozoan Parasite Phosphatomes

Comparative analysis of the phosphatomes shows that Kinetoplastid parasites, *Eh*, *Gl* and *Tv* have larger phosphatomes than Apicomplexan parasites (Fig 1). The phosphatome of *Tv* being the largest followed by *Eh* and *Tc*. Apicomplexan parasites have relatively smaller phosphatomes with maximum number of phosphatases in *Tg* followed by *Plasmodium sp*., the kinomes of these parasites are also small ranging from 35 kinases in *Bb* to 135 in *Tg* (Table 1) [21]. The phosphatomes vary in size between genera from 482 phosphatases in *Tv* to 35 in *Ec*. The phosphatome of *Eh* consists of 250 phosphatases that were reported in our earlier analysis [5, 6]. There was a huge variation in phosphatome of the genera *Trypanosoma*. The phosphatome of *Tb* strains lies in the range of 158 to 161 phosphatases, while the phosphatome of *Tc* consists of 230 phosphatases; their kinomes are composed of 176 kinases in *Tb* and 190 in *Tc* [22]. *Tc* is an intracellular parasite, it invades and reproduces in different type of cells like fibroblasts and macrophages, while, *Tb* that resides in the bloodstream of the mammalian host is a solely extracellular parasite. Since, these parasites have very distinct lifecycles, these organisms go through entirely different environments, leading to changes in expression of gene, protein levels and modification of proteins [23–25]. Protein phosphorylation actively regulates adaptive responses to intra cellular and extra cellular signals which shows marked changes during growth of these parasites [20, 26], indicating that *Tc* employs protein phosphorylation more aggressively as it needs to regulate the function of various different cell types while invading. Different species of the genus *Plasmodium* have comparatively smaller and similar phosphatomes comprising of 77 to 79 phosphatases (Table 1). In the earlier studies ratio of kinases to phosphatases in *Pf* is reported to be close to 2:1 [27], but in our analysis we have found ratio is ~1:1. Phosphatome of *Lm* has 172 phosphatases, while its kinome has 199 kinases [15], *Gl* has a phosphatome size of 151 phosphatases and its kinome consists of 278 kinases (Table 1). Human phosphatome has 199 proteins consisting of phosphatase domain [28]. There was a variation in apicomplexan phosphatomes size between different genera. Total number of phosphatases encoded in the genomes of the analyzed protozoan parasites along with the number of phosphatases reported in earlier studies [14, 15] and the percentage of phosphatome in the proteome of specific organisms is given in Fig 1. Comparison of the percentage of phosphatome in the respective proteome shows that, it varies from 1.0 to 3.1%. *Eh* has the highest percentage of phosphatases followed by *Lm* and *Cp*. The percentage of phosphatome in *Hs* is 0.6% of its proteome (Fig 1) [5, 28], which is much less than the selected parasites phosphatomes, indicating that vertebrates have various other mechanisms for the regulation of proteins but in protozoan parasites phosphorylation is an integral mechanism for protein regulation.

### 1.4 Correlation between Protozoan Parasite Proteome and Phosphatome

**Fig 2:**
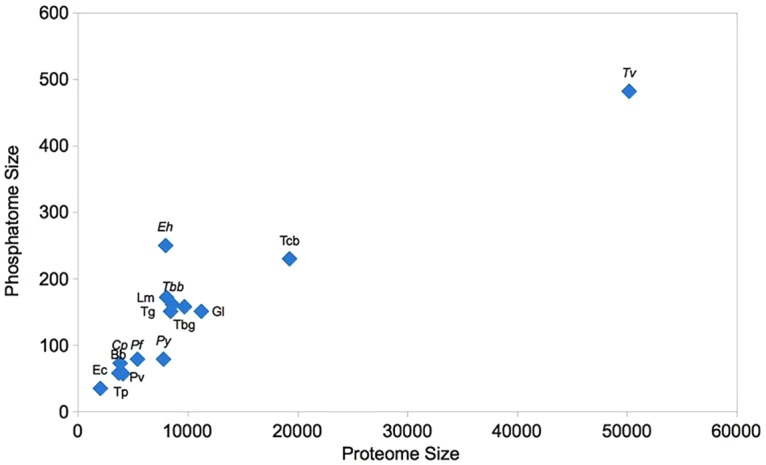
Correlation between proteome and phosphatome size of parasitic protozoa.

Analysis of the proteome size versus the phosphatome size reveals that the size of phosphatome increases with the increasing size of proteome, the only exception being *Eh*. The size of *Tc* proteome is more than double of *Eh* but still their phosphatomes are comparatively similar. A significant positive relation between phosphatome and proteome size was observed in the present study among the protozoan (Fig 2).

The size of the phosphatomes, to some extent reveals the significance of protein phosphorylation in signal transduction regulation and metabolic processes in these parasites. The difference in phosphatome size in case of *Eh* might be due to evolutionary pressure towards diversification of signal transduction pathways. The phosphatome of *Eh* is 1.3 times of human phosphatome [28]. It is striking to find that however the proteome size is highly dissimilar but their phosphatome size appear to be stable in the analyzed unicellular parasites.

### 1.5 Comparative Analysis and Distribution of Phosphatases in Protozoan Parasite

**Fig 3:**
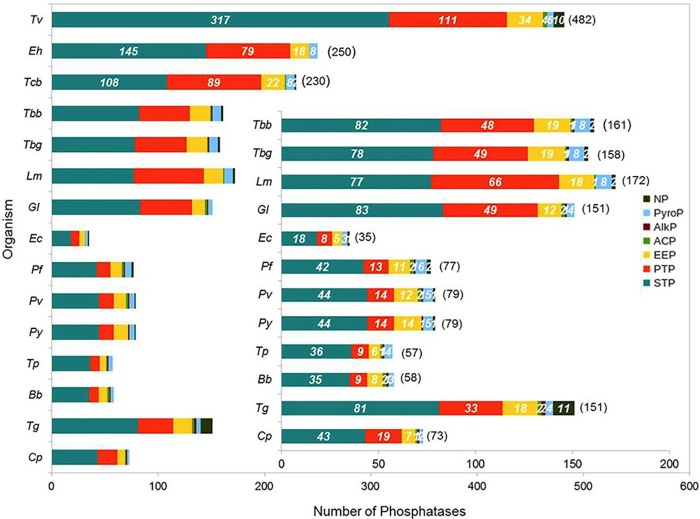
Distribution of protozoan parasite phosphatases into families of phosphatases.

Parasitic protozoan have phosphatases belonging to all the major familes of phosphatases including Serine/threonine phosphatases (STP), Protein Tyrosine Phosphatses (PTP), Endonuclease/Exonuclease/phosphatases (EEP), Acylphosphatase (ACP), Alkaline phosphatase (AlkP), Pyrophosphatases (PyroP) and Nucleoside phosphatase (NP), though their distribution vary in each protozoa (Fig 3).

The phosphatome of the analyzed parasitic protozoans is dominated by STP, revealing ser/thr phosphorylation is the most abundantly used in parasitic protozoans (Fig 3). In contrast the human phosphatome consists only 24.6% of phosphatases from STP family [28]. Ser/Thr phosphatases comprise of three unrelated sub-families Phosphoserine phosphatases (PPP) and Metal Dependent Phosphatases (PPM) and Aspartate-based phosphatases (FCP), the active sites of PPP and PPM sub-families are remarkably similar while the sub-families do not share any sequence homology, members of FCP are also ser/thr specific phosphatases [7, 29]. FCP consists aspartate based ser/thr phosphatases and haloacid dehalogeneases (HAD) [29]. Among STP’s, FCP sub-family was most represented in all the protozoans except for *Tv* and *Eh*. In Apicomplexa parasites members of the sub-family PPP and PPM were almost equivalent except for *Bb* and *Tg*. In *Eh*, *Gl* and Kinetoplastids phosphatases from the sub-family PPP were nearly double the number of PPM sub-family members. In case of *Tv* PPPs were more than double the number of FCPs, while PPMs were much less in comparison. In *Ec* also, FCPs were double that of PPPs, while PPMs were totally absent. FCP sub-family that dominated PPP phosphatome in all the parasitic protozoa was mostly composed of haloacid dehalogenases, suggesting that protozoans are generally rich in acid phosphatases.

PTP phosphatomes are relatively smaller than the STP phosphatome in all the unicellular parasites. *Tc* and *Lm* have highest percentage of PTP phosphatome, 39% and 38% respectively. Apicomplexa parasites generally have smaller PTP phosphatome (17% to 26%) in comparison to Kinetoplastids, except for *Tg* and *Cp* comprising 22% and 26% of their phosphatome, respectively. In contrary, the PTP phosphatome of humans is larger than STP phosphatome, containing 54.3% of PTPs [28]. Protein tyrosine phosphatases (PTP) consist of dual specificity phosphatase (DSPc), classical protein tyrosine phosphatase (PTPc), protein tyrosine phosphatase like (PTPLA), low molecular weight protein tyrosine phosphatase (LMPTP) and histidine phosphatase (HP) [30]. In general, PTPs from sub-families DSPc and HP were most abundant in all the parasitic protozoans, but in case of *C*. *parvum* and *T*. *parva* composition of HP were double that of DSPc. In general, PTPs from sub-families DSPc were most abundant in all the parasitic protozoans.

The investigated unicellular parasites have the large proportion of STPs and a low proportion of PTPs that is opposite to that of vertebrates having higher proportion of PTPs and lower proportion of STPs, suggesting different protein phosphorylation mechanisms in these unicellular parasites. Although PTPs comprises a smaller fraction compared to STPs, it plays an important role in signaling process regulating cell cycle control. Therefore, PTPs can possibly be potent targets for pharmacological approach.

If we look at the abundance of EEP, Kinetoplastids and *T*. *cruzi* has highest frequency of EEPs. Exonuclease-Endonuclease-Phosphatases (EEP) family consist of members from EEP sub-family and INPP sub-family. In *Trypanosoma* sp., *Plasmodium sp*. and *Eh* number of EEPs is greater than INPPs. In case of *Tv* INPPs are around 4 times more in number than EEPs. In general, fewer numbers of INPPs were observed in *Ec* and Apicomplexa parasites (Fig 3).

Pyrophosphatase in parasitic protozoans consists of inorganic phosphatases in most of the organisms (Fig 3). Earlier reports also suggest that appropriate balance of intracellular inorganic phosphate is important for cellular metabolism [31]. Membrane bound inorganic pyrophosphatases were present in all the kinetoplastids, apicomplexa (*Plasmodium sp*., *Bb*, *Tg*, *Tp*) and *Gl*. It has been reported that membrane proteins are critical targets for drugs and humans lack membrane-bound pyrophosphatases but they are vital for the survival of protozoan parasites [32], thus, the identified pyrophosphatases can be explored to understand their mechanism of action as well as these can be targeted against the parasitic organisms.

Phosphatases from several other families like acylphosphatase (ACP), alkaline phosphatase (AlkP) and nucleoside phosphatase (NP) were also observed in the analyzed unicellular parasites. Acylphosphatase is a ubiquitous small enzyme that acts on substrates like acetylphosphate, succinyl phosphate, 1,3-bisphosphoglycerate, carbamoylphosphate and *β*-aspartyl-phosphate membrane pump [33]. Acylphosphatases can be differentiated from acid phosphatases, alkaline phosphatases or protein phosphatases, as they cannot hydrolyze other phosphate esters or anhydrides. *T*. *vaginalis* has highest frequency of ACPs, others have either 1 or 2 members, while *Ec* and *Py* does not have ACPs. Alkaline phosphatase (AlkP) has the ability to remove phosphate groups from several kinds of molecules, such as proteins, nucleotides and alkaloids; at least one member was present in all the Apicomplexa parasites. In *Ec*, *Tv*, *Tc* and *Lm* AlkPs were not seen, while in *Tb* strains and *Gl* a single member is found. Largest number of NPs was seen in *Tg* followed by *Tv*. *Trypanosoma* sp., *Plasmodium sp*., *Lm* and *Ec* have 1 or 2 members of NPs, while it is absent in *Gl*, *Tp*, *Cp* and *Bb* (Fig 3, Table A in S1 File).

Large differences in the phosphatomes between different genera of parasitic protozoans indicate considerable variability in the signaling pathways they are involved in. The phosphatome of humans is dominated by PTPs in contrary to the unicellular parasites having STPs in abundance [23].

### 1.6 Uncharacterized Phosphatatome of Protozoan Parasites

**Fig 4:**
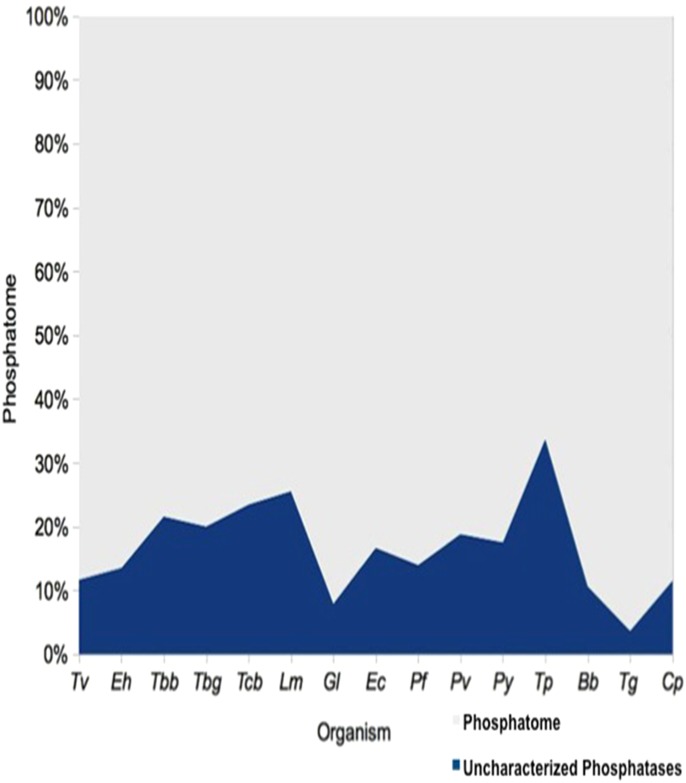
Percentage of uncharacterized proteins classified as phosphatases on the basis of domain identification.

In the present study we have analyzed 14 unicellular parasites and identified ~2000 phosphatases, out of which 21% are uncharacterized proteins. Highest percentage of uncharacterized proteins that were assigned to different families of phosphatases belongs to *Tp* (51.0%), followed by *Lm*, *Tc* and *Tbb* (Fig 4). For the sake of comparison we have taken the *Eh* phosphatome data that was analyzed in our previous work [5, 6]. A list of number of uncharacterized phosphatases classified as phosphatases in each genome is shown in Table B in S1 File.

### 1.7 Subcellular Localization of Phosphatases in Protozoan Parasites

Phosphatases can be characterized on the basis of their location in the cell, which can be intracellular i.e., secreted to the extracellular medium from the cell or membrane bound i.e., associated to plasma membrane [34, 35]. Phosphatases were found to be widely distributed throughout the cell (cytoplasm, mitochondria, nucleus, extracellular, plasma membrane, etc.). Highest distribution of phosphatases in the protozoans was seen in nuclear, plasma membrane and cytoplasmic regions (Table 2). Phosphatases were seen widely distributed of on the cell surface suggesting some physiological adaptation of the parasite to regulate its host. The details of the subcellular localization of each phosphatase can also be seen in ProtozPhosDB.

**Table 2:** Percentage of phosphatases distributed to different locations of the cell in protozoan parasites.

Sub-cellular Localization	*Lm*	*Tbb*	*Tbg*	*Tc*	*Pf*	*Pv*	*Py*	*Tg*	*Cp*	*Tp*	*Bb*	*Eh*	*Gl*	*Tv*	*Ec*
	Kinetoplastids				Apicomplexa							Others			
Cytoplasmic	23.3	29.8	27.8	25.2	14.3	16.5	7.6	21.2	20.5	33.3	31.0	30.7	28.5	38.6	48.6
Mitochondrial	11.6	11.2	11.4	13.0	5.2	5.1	8.9	6.0	2.7	−	3.4	0.4	3.3	3.7	−
Nuclear	32.0	26.1	26.6	19.1	54.5	54.4	48.1	47.7	46.6	26.3	25.9	33.9	21.9	23.9	20.0
Extracellular	5.8	6.2	5.7	5.7	−	3.8	−	2.6	−	1.8	−	1.6	1.3	4.6	−
Plasma Membrane	21.5	24.2	24.1	29.6	26.0	20.3	31.6	17.2	27.4	38.6	39.7	32.7	43.7	28.2	28.6
Glycosome	0.6	0.6	−	−	−	−	−	−	−	−	−	−	−	−	−
Vacuole	−	−	−	−	−	−	−	−	−	−	−	−	−	0.2	−
Uncharacterized	5.2	1.9	4.4	7.4	−	−	3.8	5.3	2.7	−	−	0.8	1.3	0.8	2.9

### 1.8 Molecular Function, Biological Process & Pathways Protozoan Parasite Phosphatases are Involved

**Fig 5:**
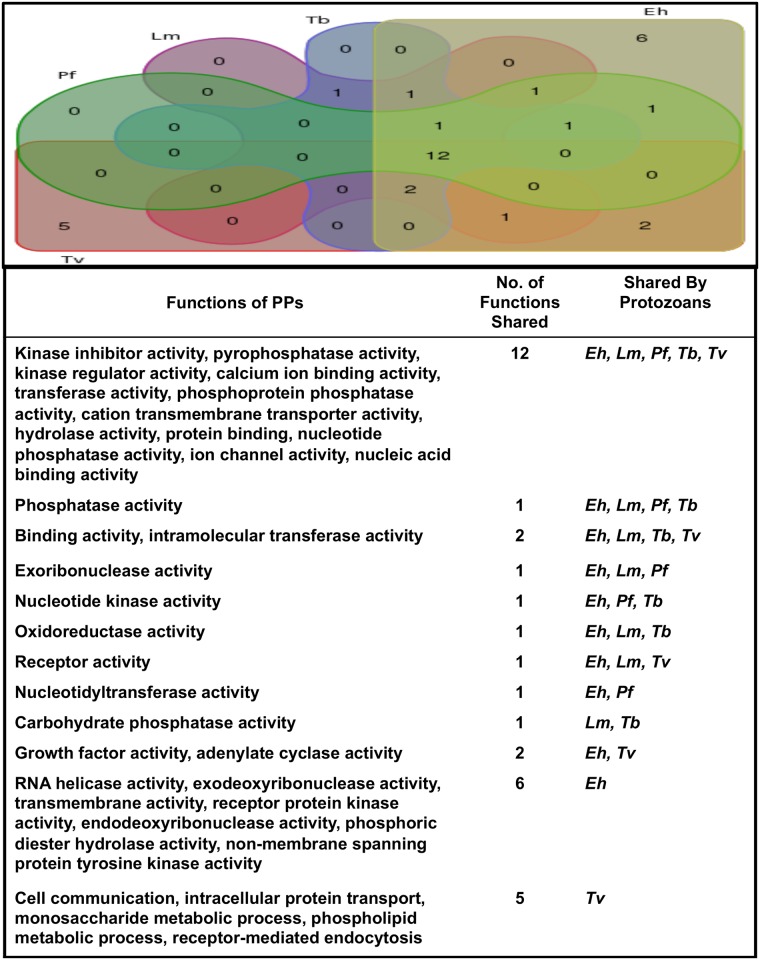
Venn diagram representing the analyses of conserved and specific functions of phosphatases in protozoan parasite.

Phosphatases are involved in plethora of biological functions. The functional analysis data of whole genomes was available for only 5 protozoans (*Pf*, *Lm*, *Tb*, *Eh* and *Tv*). We have seen that phosphatases were involved in 34 distinct functions. Kinase inhibitor activity, pyrophosphatase activity, kinase regulator activity, calcium ion binding activity, transferase activity, phosphoprotein phosphatase activity, cation transmembrane transporter activity, hydrolase activity, protein binding, nucleotide phosphatase activity, ion channel activity, nucleic acid binding activity were found to be conserved in all the analyzed protozoans. Several functions like RNA helicase activity, exodeoxyribonuclease activity, transmembrane activity, receptor protein kinase activity, endodeoxyribonuclease activity, phosphoric diester hydrolase activity, non-membrane spanning protein tyrosine kinase activity were specific to *Eh* only. Phosphatases having specific function in addition to the conserved functions, justifies the higher number of phosphatases in *Eh*. Some functions were specific to *Tv* only including cell communication, intracellular protein transport, monosaccharide metabolic process, phospholipid metabolic process, receptor-mediated endocytosis (Fig 5).

### 1.9 Ectophosphatases in Protozoan Parasites

Parasites come across various membranes and secreted molecules for attachment and invasion of the host cells. Ectophosphatases are potentially important phosphatase group, which is the phosphatase associated with the outer cell surface having their active sites oriented towards the external medium rather than the cytoplasm [36–38]. Ectophosphatases play an important role in cell proliferation, differentiation, parasite-host interaction, nutrition, ROS sensing and adhesion [39, 40]. Ectophosphatases have been reported in some protozoa parasites, including *Tb*, *Tc*, *Eh*, *Gl*, *Tv* and some *Leishmania* species [8, 39, 41–52]. A unique ectophosphatase cloned from *Tb* does not have any other similar protein beyond the genus *Trypanosoma* [1]. In the present analysis the phosphatases localized in the extracellular space and plasma membrane were considered as ectophosphatases and we found that 20 to 40 percent of the phosphatomes of different unicellular parasites consists of ectophosphatases (Table 2). In most of the organisms ectophosphatases are dominated by the members from HAD sub-family, though ectophosphatases consist of phosphatases from all the representative families. Earlier reports also suggest that the surface localization and low optimum pH of acidic phosphatases helps to function in acidic microenvironment [53–55], perhaps reflecting an adaptation to the extracellular environment by the parasite.

## Conclusions

The genome sequence of parasitic protozoans provides a greater understanding of protein regulation and potential avenues for drug therapies and vaccine development. Lot of work has been done on the functional characterization of protein kinases; a better understanding of phosphatases and their involvement in parasite physiology and the identification of potential inhibitors to disrupt parasitic function are essential. This study provides the first account of our current understanding of protein phosphatomes of protozoan parasites having completed genomic sequences. The phosphatome size is positively correlated with the proteome size except for *Eh*. This may be due to extensive use of protein phosphorylation for regulation of proteins by *Eh*, while rest of the parasitic protozoans deploy other mechanism too. Thus, despite of having a small phosphatome, the parasite manages to maintain a large functional proficiency. The overall distribution of phosphatases within different protozoa genus seems to differ greatly. The distribution of phosphatases in different species of *Trypanosoma* was slightly variable, while *Plasmodium* sp. was having similar distribution of phosphatases. The differences in the phosphatomes of these parasites may be due to the variation in the living environments in which they exist. Interestingly, the phosphatome of these parasites have larger number of STPs unlike vertebrate host that is dominated by PTPs. Ectophosphatases apart from hydrolyzing extracellular phosphorylated substrates to change their function also have several alternative functions, such as adhesion, differentiation, participation in proliferation, virulence and infection. The identified ectophosphatases can be analyzed to understand the physiological roles of these enzymes in protozoan parasites. Further, ectophosphatases can be promising new drugs targets for treatment of these parasitic infections. Uncharacterized proteins are the loopholes in our understanding of biological processes. The dataset of uncharacterized proteins identified, as phosphatases can be further analyzed to understand physiological roles of these enzymes in the regulatory network of protozoan parasites. ProtozPhosDB can be used to access any information related to phosphatases in protozoans of medical and veterinary importance worldwide. The phosphatome data can be used to design experimental work to interpret the function of individual phosphatase in any particular parasitic protozoa and the signal transduction pathways they are involved in.

## Supporting Information

S1 File *Table A*. Distribution of protozoan parasite phosphatases into families and sub-families of phosphatases. *Table B*. Distribution of protozoan parasite uncharacterized proteins into phosphatase families.
(DOC)
