# Supplementary material for: Deep Insight into the Phosphatomes of Parasitic Protozoa and a Web Resource ProtozPhosDB
Source: PLoS One. 2016 Dec 8;11(12):e0167594. doi: 10.1371/journal.pone.0167594 (PMC5145157; doi:10.1371/journal.pone.0167594)
Supplement: S1 File — Table A. Distribution of protozoan parasite phosphatases into families and sub-families of phosphatases. Table B. Distribution of protozoan parasite uncharacterized proteins into phosphatase families. (DOC) [file pone.0167594.s001.doc]

**Table A**. Distribution of protozoan parasite phosphatases into families and sub-families of phosphatases.

| **Organism** | **STP** | | | **PTP** | | | | | **EEP** | | **ACP** | **AlkP** | **PyroP** | **NP** | **Total** |
| --- | --- | --- | --- | --- | --- | --- | --- | --- | --- | --- | --- | --- | --- | --- | --- |
|  | **PPP** | **PPM** | **FCP** | **DSPc** | **HP** | **PTPLA** | **PTPc** | **LMWPTP** | **EEP** | **INPP** |  |  |  |  |  |
| ***C. parvum*** | **10** | **14** | **19** | **6** | **11** | **1** | **1** |  | **4** | **3** | **1** | **1** | **2** |  | **73** |
| ***T. gondii*** | **11** | **39** | **31** | **14** | **15** | **1** | **3** |  | **10** | **8** | **2** | **2** | **4** | **11** | **151** |
| ***B. bovis*** | **13** | **2** | **20** | **4** | **4** | **1** |  |  | **4** | **4** | **2** | **1** | **3** |  | **58** |
| ***T. parva*** | **8** | **5** | **23** | **3** | **6** |  |  |  | **3** | **3** | **1** | **1** | **4** |  | **57** |
| ***P. yoeli*** | **11** | **13** | **20** | **6** | **6** | **1** | **1** |  | **10** | **4** |  | **1** | **5** | **1** | **79** |
| ***P. vivax*** | **13** | **13** | **18** | **6** | **6** | **1** | **1** |  | **8** | **4** | **2** | **1** | **5** | **1** | **79** |
| ***P. falciparum*** | **12** | **12** | **18** | **5** | **6** | **1** | **1** |  | **7** | **4** | **2** | **1** | **6** | **2** | **77** |
| ***L. major*** | **33** | **16** | **28** | **34** | **22** | **1** | **8** | **1** | **9** | **9** | **1** |  | **8** | **2** | **172** |
| ***T. b. brucei*** | **30** | **15** | **37** | **26** | **14** | **1** | **6** | **1** | **11** | **8** | **1** | **1** | **8** | **2** | **161** |
| ***T. b. gambiens*** | **29** | **15** | **34** | **25** | **16** | **1** | **6** | **1** | **10** | **9** | **1** | **1** | **8** | **2** | **158** |
| ***T. cruzi*** | **32** | **19** | **57** | **46** | **30** | **1** | **11** | **1** | **13** | **9** | **1** |  | **8** | **2** | **230** |
| ***E. histolytica*** | **64** | **42** | **39** | **36** | **19** | **1** | **21** | **2** | **11** | **7** |  |  | **8** |  | **250** |
| ***T. vaginalis*** | **205** | **26** | **86** | **45** | **49** |  | **9** | **8** | **7** | **27** | **4** |  | **6** | **10** | **482** |
| ***G. lamblia*** | **35** | **12** | **36** | **25** | **15** | **5** | **2** | **2** | **2** | **10** | **2** | **1** | **4** |  | **151** |
| ***E. cuniculi*** | **6** |  | **12** | **2** | **4** | **1** | **1** |  | **2** | **3** |  |  | **3** | **1** | **35** |

*List of abbreviations used for phosphatase families

Serine/threonine phosphatases (STP), Phosphoserine phosphatases (PPP), Metal Dependent Phosphatases (PPM), Aspartate-based phosphatases (FCP), Protein Tyrosine Phosphatses (PTP), Dual specificity phosphatase (DSPc), Histidine phosphatase (HP), Protein tyrosine phosphatase like (PTPLA), Classical protein tyrosine phosphatase (PTPc), Low molecular weight protein Tyrosine phosphatase (LMPTP), Endonuclease/Exonuclease/phosphatases (EEP), Inositol polyphosphate 1-phosphatase (INPP), Acylphosphatase (ACP), Alkaline phosphatase (AlkP), Pyrophosphatases (PyroP) and Nucleoside phosphatase (NP)

**Table B**. Distribution of protozoan parasite uncharacterized proteins into phosphatase families.

| **Organism** | **STP** | **PTP** | **EEP** | **ACP** | **AlkP** | **PyroP** | **NP** | **Total** |
| --- | --- | --- | --- | --- | --- | --- | --- | --- |
| ***C. parvum*** | **4** | **3** | **2** | **-** | **-** | **1** | **-** | **10** |
| ***T. gondii*** | **2** | **3** | **-** | **1** | **-** | **-** | **-** | **6** |
| ***B. bovis*** | **3** | **2** | **1** | **1** | **-** | **-** | **-** | **7** |
| ***T. parva*** | **17** | **4** | **5** | **-** | **1** | **2** | **-** | **29** |
| ***P. yoeli*** | **9** | **6** | **1** | **-** | **-** | **-** | **1** | **17** |
| ***P. vivax*** | **9** | **5** | **2** | **1** | **1** | **1** | **-** | **19** |
| ***P. falciparum*** | **6** | **4** | **1** | **1** | **1** | **-** | **-** | **13** |
| ***L. major*** | **30** | **26** | **4** | **-** | **-** | **1** | **-** | **61** |
| ***T. b. brucei*** | **22** | **20** | **3** | **-** | **1** | **1** | **-** | **47** |
| ***T. b. gambiens*** | **20** | **17** | **3** | **-** | **1** | **1** | **-** | **42** |
| ***T. cruzi*** | **31** | **35** | **6** | **-** | **-** | **3** | **-** | **75** |
| ***E. histolytica*** | **21** | **14** | **2** | **-** | **-** | **2** | **-** | **39** |
| ***T. vaginalis*** | **41** | **10** | **2** | **3** | **-** | **-** | **10** | **66** |
| ***G. lamblia*** | **5** | **8** | **-** | **-** | **-** | **-** | **-** | **13** |
| ***E. cuniculi*** | **5** | **2** | **-** | **-** | **-** | **-** | **-** | **7** |
